# Asynchronous actions of immune responses in COVID-19 patients

**DOI:** 10.1038/s41392-020-00424-z

**Published:** 2020-12-04

**Authors:** Yufang Shi, Guoqiang Zhou, Qing Li

**Affiliations:** 1 The First Affiliated Hospital of Soochow University and State Key Laboratory of Radiation Medicine and Protection, Institutes for Translational Medicine, Soochow University, 199 Renai Road, Suzhou, Jiangsu 215123 China; 2 CAS Key Laboratory of Tissue Microenvironment and Tumor, Shanghai Institute of Nutrition and Health, University of Chinese Academy of Sciences, Chinese Academy of Sciences, 320 Yueyang Road, Shanghai, 200030 China; 3 Department of Gastrointestinal Surgery, the Second People’s Hospital of Changshu, the Affiliated Changshu Hospital of Xuzhou Medical University, 68 Haiyu Road, Changshu, 215500 China

**Keywords:** Adaptive immunity, Infection

Various components of the immune system are fine-tuned and coordinated for the protection and maintenance of tissue homeostasis in various organs. Evolution has ensured the precise activation and synchronization of specific immune components at a given time to provide proper defense and regeneration environment to tissues of our body. Any disorders may lead to imperfect protection and uncontrolled tissue damages. A new study from the Sette and Crotty group just reported in Cell demonstrated that COVID-19 patients, especially at elder ages, suffered the most when the immune components were asynchronous.^1^

**Fig. 1:**
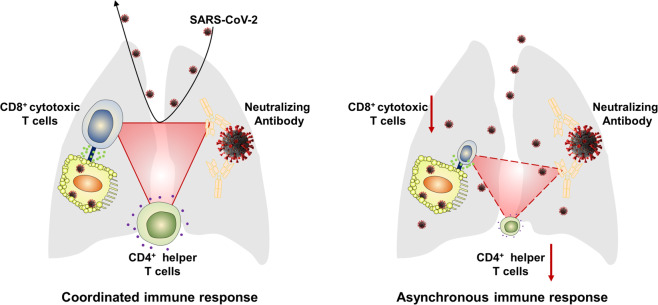
SARS-CoV-2 antigen-specific CD8+ T cells, CD4+ T cells, and neutralizing antibodies. **a** Troika combating COVID-19 infection. A coordinated response involving CD8^+^ cytotoxic T cells, CD4^+^ helper T cells, and neutralizing antibodies is required to provide the best protective effects for SARS-CoV-2 infections. However, in older patients whose CD8^+^ T and CD4^+^ T cell responses are compromised, the diseases are prone to progress to severe stages

Like most viral infections, SARS-CoV-2 elicits strong inflammatory responses. It has been proposed that the immune responses induced by SARS-CoV-2 infection are two-phased. Specific adaptive immune responses are critical for the elimination of the virus during the incubation and non-severe stages and for stopping disease progression to severe stages. When the protective immune responses are insufficient, due to genetic predisposition or preexisting medical conditions, the virus will propagate and massive destruction of the affected tissues, such as lungs, will occur.^2^ Most studies are focused on the inflammation and tissue damages at severe stages. However, recent investigations on non-severe or convalescent patients have provided clues to the understanding of protective immune responses.

In an effort to understand human CD4^+^ and CD8^+^ T cell responses to SARS-CoV-2 infection, the same research group employed human leukocyte antigen (HLA) class I and II predicted peptide “megapools”^3^ and CD8^+^ and CD4^+^ T cells from convalescent patients and found that 87% and 93% of these recovered patients possessed SARS-CoV-2-specific CD8^+^ and CD4^+^ T cells, respectively.^1^ Remarkably, when antibodies (Abs) made from B cell clones derived from convalescent patients were tested for their ability to neutralize SARS-CoV-2 virus in vitro in plastic and in vivo in hamsters, it was found that the receptor binding domain (RBD) specific Ab provided strong protection.^4^

Upon until now, the most comprehensive study on SARS-CoV-2 virus-specific adaptive immunity in humans is by the Sette and Crotty group just published in *Cell*, which is the subject highlighted herein. It not only involved a larger number of convalescent and acutely infected patients, but also divided adaptive immune responses into viral protein-specific CD4^+^ T cells, CD8^+^ T cells, and Abs, the three most critical components of the adaptive immune responses. Although each component can work separately, together, the three components bring best protection. In the vaccinia virus system, based on the pooled viral peptides, it was shown that CD8^+^ T cell, CD4^+^ T cell, and Ab responses tend to recognize different antigens with distinct characteristics, CD8^+^ T cells recognize early antigens, and CD4^+^ T cells and Abs recognize later antigens.^5^ This same pattern is also demonstrated by Moderbacher et al. in SARS-CoV-2 infection, during which all three arms work together to bring the best protection and if these adaptive immune responses are not synchronized COVID-19 patients are in trouble.^1^ In this coordinated process, B cell-produced Abs are able to attach to and neutralize extracellular SARS-CoV-2 virus. For various reasons, if the Abs are unable to stop the virus from entering cells, CD8^+^ T cells are called in to destroy the infected cells. Regarding CD4^+^ T cells, the third arm, they are helpers and coordinators for production of Abs and the activation of CD8^+^ killer T cells (Fig. 1).

Moderbacher et al. analyzed the blood of COVID-19 patients suffering from mild to ultimately fatal infection. Their immune responses were compared to those of convalescents and unexposed control individuals. The Ab levels alone did not correlate with COVID-19 severity. Those worst cases of COVID-19 had low levels of CD8^+^ killer T cells and CD4^+^ T helper cells. It is highly possible that T cells play a more important role than Abs in combating ongoing COVID-19 infections. In fact, the authors identified one case that had no detectable neutralizing antibodies and resolved infection without hospitalization. In addition, some infected children recovered before developing an Ab response, again arguing for the importance of T cells.

When blood samples from the older participants (≥65 years old) with acute infections were analyzed, it was found that they were far more likely to have asynchronous immune responses among CD4^+^ T cells, CD8^+^ T cells, and Abs than younger infected patients. In older patients, high levels of Abs could be seen, while one of the T cell responses remained weak. Interestingly, older COVID-19 patients tended to also have smaller populations of the “naive” T cells, which could recognize the new SARS-CoV-2 virus and then develop into mature CD8^+^ killer T cells and CD4^+^ T helpers, which otherwise mounts a coordinated attack against SARS-CoV-2.

There is much talk about the cytokine storm in COVID-19 patients. It should be recognized that due to the lymphopenia in severe patients, the cytokine storm in these patients should not be expected to be the same as that observed in other patients such as chimeric antigen receptor T cell recipients. In the lack of T cells, the antibody response is unable to constrain SARS-CoV-2 and allows the virus to replicate to high levels. The innate immune cells release high levels of cytokines, which, in the absence of T cell cytokines, further damage the adaptive immunity and vital organs such as the lungs, kidneys, and cardiovascular system. It should be clearly recognized that such a cytokine storm without T cells is distinctly different from that in other overreactions of the immune system during infections or some other pathological conditions. Although the mechanisms underlying the diminishment of T cells in severe COVID-19 patients are not known, it is clear that immunity-damaging agents such as dexamethasone should be used cautiously, especially during the early phase of the infections, when adaptive immune responses are required to prevent the progression of the disease.

The information from the analysis of Moderbacher et al. also provided important clues to vaccine development.^1^ T cell responses should be accentuated. So far, COVID-19 vaccine development efforts are mostly focused on the S protein of SARS-CoV-2 with the hope to induce SARS-CoV-2 neutralizing antibodies. Ironically, only antibodies against the RBD region of the S protein provide strong virus neutralization. Based on the requirement of the synchrony of CD8^+^ T cells, CD4^+^ T cells, and Abs, effective vaccines should elicit both antibody and T cell responses and strong T cell responses may require M proteins and NP proteins in addition to S proteins. Additionally, T cell responses should also be used to evaluate SARS-CoV-2 vaccines.

This is one of the most comprehensive analyses of SARS-CoV-2-specific immunity in patients. It provided critical information for the understanding of how to stop disease progression and how to design efficacious treatments and how to develop better vaccines.

## References

[1] Rydyznski Moderbacher C (2020). Antigen-specific adaptive immunity to SARS-CoV-2 in acute COVID-19 and associations with age and disease severity. Cell.

[2] Shi Y (2020). COVID-19 infection: the perspectives on immune responses. Cell Death Differ..

[3] Grifoni A (2020). Targets of T cell responses to SARS-CoV-2 coronavirus in humans with COVID-19 disease and unexposed individuals. Cell.

[4] Rogers TF (2020). Isolation of potent SARS-CoV-2 neutralizing antibodies and protection from disease in a small animal model. Science.

[5] Moutaftsi M (2010). Uncovering the interplay between CD8, CD4 and antibody responses to complex pathogens. Future Microbiol..

